# Early versus delayed hip reduction in the surgical treatment of femoral head fracture combined with posterior hip dislocation: a comparative study

**DOI:** 10.1186/s12891-021-04968-1

**Published:** 2021-12-20

**Authors:** Shanxi Wang, Bohua Li, Zhengdong Zhang, Xiaojun Yu, Qin Li, Lei Liu

**Affiliations:** 1 Department of Orthopedics, Tongji Hospital, Tongji Medical College, Huazhong University of Science and Technology, Jiefang Avenue 1095, Wuhan, 430030 People’s Republic of China; 2 Department of Orthopedics, West China Hospital, Sichuan University, 37# Guoxue Alley, Chengdu, 610041 Sichuan People’s Republic of China

**Keywords:** Femoral fractures, Posterior hip dislocation, Early reduction, Reduction timing

## Abstract

**Background:**

Few studies focus on the treatment of femoral head fracture combined with posterior hip dislocation, and the safe interval time between injury and hip reduction remains controversial. The purpose of this study was to evaluate and compare the outcome of early and delayed hip reduction in the surgical treatment of femoral head fracture combined with posterior hip dislocation.

**Methods:**

A total of 71 patients were evaluated in this retrospective study. Based on the time to hip reduction, they were divided into early group (within 6 h after injury) and delayed group (between 6 and 12 h after injury). The two groups were compared in reference to hospital day, fracture healing time, the occurrence of complications and final functional outcome. The Thompson-Epstein criteria, modified Merle D’Aubigné and Postel scores, visual analog scale (VAS) and Medical Outcomes Short Form 12-item questionnaire score (SF-12) were used for final functional evaluation.

**Results:**

The mean hospital stay and fracture healing time in the early group were significantly lower than those in the delayed group. The incidence of infection, post-traumatic osteoarthritis, and avascular necrosis of the femoral head (ANFH) in the delayed group were higher than that in the early group. The early group had better functional outcomes in terms of Thompson-Epstein criteria, modified Merle D’Aubigné and Postel scores and physical component scale (PCS) than the delayed group.

**Conclusions:**

For the treatment of femoral head fracture combined with posterior hip dislocation, the early and prompt hip reduction can effectively facilitate the fracture healing and patient rehabilitation, and obtain a better functional outcome.

## Background

Femoral head fractures are relatively infrequent injury, occurring often following traumatic posterior hip dislocation, and it make up 4 to 17% of posterior hip dislocation [1–3]. In 1957, Pipkin proposed the Pipkin classification of femoral head fractures combined with posterior hip dislocation, which is based on the location of the fracture line relation to the fovea and the potential presence of the femoral neck or acetabulum [4]. Because of the widely used of Pipkin classification in clinical works, it has greatly promoted the understanding of femoral head fractures, thus the femoral head fractures combined with posterior hip dislocation is also called Pipkin fractures [5].

Owing to the complexity of the hip anatomy, the treatment of femoral head fractures is particularly difficult. Although some studies have reported satisfactory results of nonsurgical treatment in patients with non-displaced Pipkin type I and type II fractures, this treatment has almost been abandoned because of the high rate of complications associated with longstanding patient immobility and the high cost of prolonged admission [1, 6–9]. And more and more investigators recommend surgical treatment of femoral head fractures [1–3, 8, 10–13].

For the treatment of femoral head fractures combine with posterior hip dislocation, the early and prompt hip reduction is associated with a good result [1, 2]. However, because of the low incidence and limited numbers of patients, there is still no consensus on the reduction timing of posterior hip dislocation in the treatment femoral head fracture-dislocations, and the reduction timing between injury and hip reduction is different in previous studies [2, 14]. Although it has been reported that the good results can be achieved when posterior hip dislocation reduction was reduced within 12 h or 24 h, some authors emphasize that the posterior dislocation of hip should be reduced within 6 h [7, 15–17]. The purpose of this study was to compare the outcomes of early and delayed hip reduction in the surgical treatment of femoral head fracture combined with posterior hip dislocation, and to define the best timing reduction.

## Patients and methods

### Study design

After obtaining approval from our institutional review board, we retrospectively reviewed patients who suffered from femoral head fractures combined with posterior hip dislocation between July 2009 and March 2017. The inclusion criteria were as follow: (a) unilateral femoral head fracture combined with posterior hip dislocation, (b) patients were treated operatively, (c) no severe neurovascular injury, (d) closed fractures. Exclusion criteria included femoral head fracture combined with anterior or central hip dislocation, pathological fracture, other fractures affecting the limb rehabilitation, polytrauma and patients were treated with non-surgical treatment.

### Interventions

All patients presented to our emergency department and then were assessed according to the Adult Trauma Life Support (ATLS™) guidelines, including a hip anteroposterior radiograph and three-dimensional computed tomography examination. The emergency closed or open reduction of posterior hip dislocation was performed under general anesthesia in operating room. After the successful reduction of posterior hip dislocation, the skeletal traction as essential to maintain the reduction, and the patients were treated operatively within 72 h.

All surgeries were performed under general anesthesia by one surgical team consisting of 2 senior orthopedics surgeons. The modified Heuter anterior approach or posterior Kocher-Langenbeck approach as previously described was chosen for Pipkin type I and type II fractures, and the Kocher-Langenbeck approach was applied for Pipkin type III and type IV fractures [9, 18]. The fractures reduction was performed under intraoperative fluoroscopy, small or comminuted fragments of the femoral head were removed and the large fragments or fragments within the weight-bearing portion were reduced anatomically and fixed with bioabsorbable screws or cannulated screws. For the Pipkin type III femoral head fractures, the patients were treated with open reduction and internal fixation initially, and the femoral neck fractures were fixed with cannulated screws. For the Pipkin type IV femoral head fractures, the large fragments were reduced and fixed with reconstruction plates plus screws, and the small and comminuted intra-articular fragments were removed.

After operation, the prophylactic intravenous antibiotics were administered for 24 h, and low molecular weight heparin were given to prevent deep venous thrombosis. The drainage was maintained for 24–48 h and then was removed. Limb functional exercise was encouraged after recovery from anesthesia. All patients were instructed to non-weight bearing for 6 to 8 weeks initially, and then gradually increased to partial weight-bearing. Once the radiographs showed bone union, full weight bearing was started. All patients would be followed at monthly until the radiographic bony union, and then at annually until the last follow-up. Serial radiographs were obtained at every follow up, and the complication were recorded.

### Outcome measures

The clinical outcome included hospital stay, fracture healing time, complications and final functional evaluation. The major complications include wound infection, post-traumatic osteoarthritis, heterotopic ossification (HO), avascular necrosis of the femoral head (ANFH) and nonunion. The hip functional outcomes were assessed according to the Thompson-Epstein criteria [16] and modified Merle D’Aubigné and Postel scores (Chinese Version) [19], the pain level was evaluated with the visual analog scale (VAS) [20] and the Medical Outcomes Short Form 12-item questionnaire score (SF-12) [21] was used for the assessment of health status. Fracture healing time, functional evaluation and complications was assessed clinically and radiographically. To avoid examiner bias, postoperative follow-up and evaluations were conducted by two surgeons who were not participated in the treatment of patients.

### Statistical analysis

All data management and statistical analysis were performed with Statistical Package for the Social Sciences (SPSS 20.0, IBM, New York City, USA). Categorical data were tabulated with frequencies or percentages, and continuous data were expressed as the mean ± standard deviation (SD). Normality was tested using the Kolmogorov-Smirnov test. Independent t-tests were used for normally distributed continuous data and the Mann-Whitney test was used to compare abnormally distributed continuous data between two groups. Chi-square test or Fisher exact test was used to analyze the categorical variables. The level of significance was set at *p* < 0.05.

## Results

### Baseline characteristics

A total of 71 patients were evaluated in this retrospective study, the posterior hip dislocation was reduced within 6 h after injury in 39 patients (early group), and 32 patients were reduced between 6 and 12 h after injury (delayed group). Follow up data for all patients were available. The posterior hip dislocation was reduced by closed reduction in 63 patients, and 8 patients required open reduction. The mean time between injury to the reduction of posterior hip dislocation in early group was significantly lower than the delayed group. There were no statistically significant differences between the two groups in terms of Pipkin classification, age, gender, side, causes, reduction method of dislocation, surgical approach, operative time, blood loss and follow-up duration. The baseline characteristics of the patients were showed in Table 1.

**Table 1:** Comparison of the baseline data of the patients between the two groups

	Early group (*n* = 39)	Delayed group (*n* = 32)	*p* value
Pipkin classification			0.986
Pipkin type I	7	7	
Pipkin type II	16	12	
Pipkin type III	6	5	
Pipkin type IV	10	8	
Age (years)	42.6 ± 13.6	39.2 ± 12.7	0.275
Gender (male/female)	24/15	24/8	0.309
Side(right/left)	21/18	15/17	0.637
Causes			0.665
Traffic accident	29	23	
Falling from height	5	5	
Heavy pound injury	3	4	
Sport injury	2	0	
Reduction method			0.454
Closed reduction	36	27	
Open reduction	3	5	
Time to reduction(hours)	4.2 ± 1.2	10.0 ± 1.6	<0.001
Surgical approach^a^			
Modified Heuter approach	15	10	0.531
Kocher-Langenbeck approach	8	9	
Operative time (min)	146.5 ± 48.0	147.3 ± 54.6	0.945
Blood loss (ml)	305.6 ± 179.6	350.7 ± 214.3	0.338
Follow-up duration (months)	66.3 ± 8.4	67.9 ± 9.0	0.420

^a^Pipkin type I and type II fractures

### Clinical outcomes

Patients in the delayed group needed a longer hospital stay than patients in the early group. The fracture healing time of delayed group was also longer than that in the early group. In the early group, no infection occurred, and the wound healed well. The post-traumatic osteoarthritis was observed in four patients, and was treated with painkiller. Five patients developed HO, and no patient elected to undergo surgical excision of the ectopic bone. ANFH occurred in two patients and nonunion occurred in one patient. These three patients chose total hip arthroplasty (THA) due to the unbearable pain and the limitation of hip function. In the delayed group, the wound infection was occurred to one case, which was superficial infections. The bacterial culture was *pseudomonas aeruginosa*, and the wound infection was cured after changing of dressing and antibiotic treatment. The post-traumatic osteoarthritis was occurred to six patients, all of them chose nonsurgical treatment. Four patients developed HO, one patient (Brooker type IV) chose surgical excision of the ectopic bone because of the limited hip flexion. ANFN was observed in five patients and nonunion was observed in one patient, these six patients also underwent THA because of the failure of nonsurgical treatment (Table 2).

**Table 2:** Comparison of hospital stay, time to bone union, complications and reoperation rate between the two groups

	Early group (*n* = 39)	Delayed group (*n* = 32)	*p* value
Hospital stay (days)	12.7 ± 3.9	14.7 ± 2.7	0.019
Fracture healing time (weeks)	13.1 ± 2.3	14.8 ± 3.0	0.008
Complications (%)			
Wound infection	0(0%)	1(3.1%)	0.451
Post-traumatic osteoarthritis	4(10.3%)	6(18.8%)	0.496
HO	5(12.8%)	4(12.5%)	1.000
ANFH	2(5.1%)	5(15.6%)	0.231
Nonunion	1(2.6%)	1(3.1%)	1.000

*HO* heterotopic ossification, *ANFH* avascular necrosis of the femoral head

**Fig.1:**
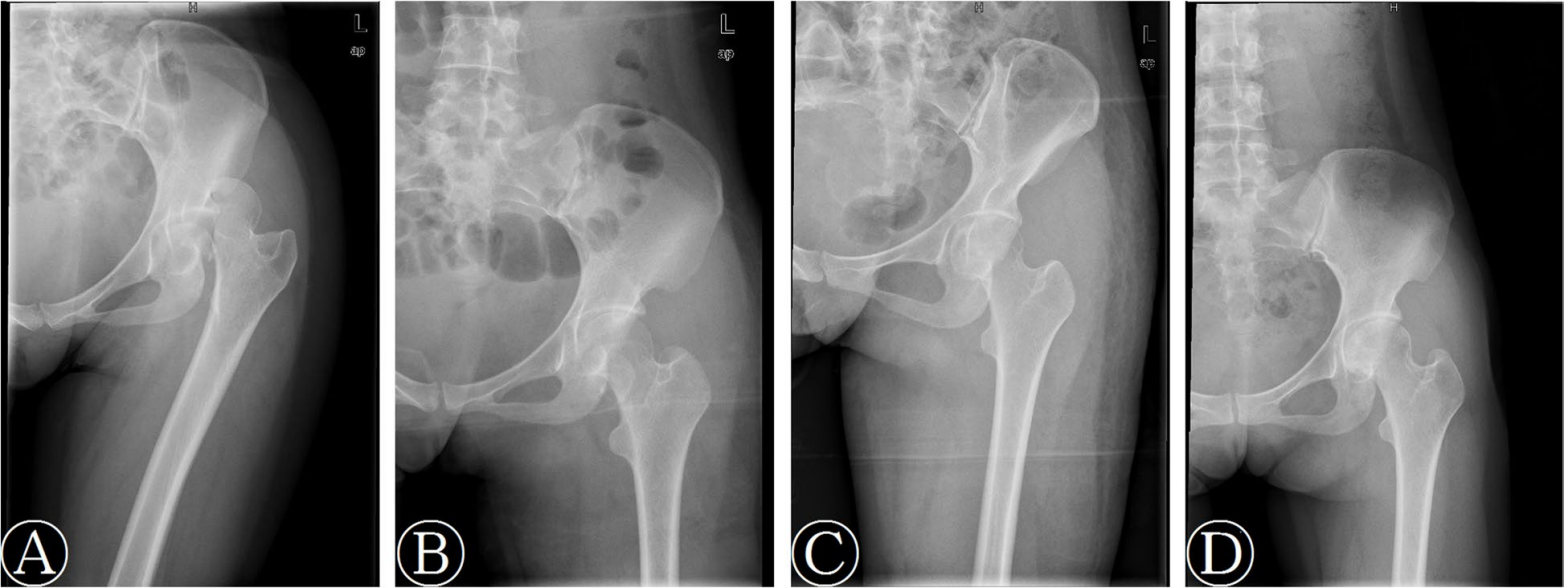
A 30-year-old woman with left Pipkin type II fracture. **A** Radiograph before reduction. **B** Radiograph after the reduction of posterior hip dislocation. **C**-**D** Post-operative and final follow-up radiographs

**Fig. 2:**
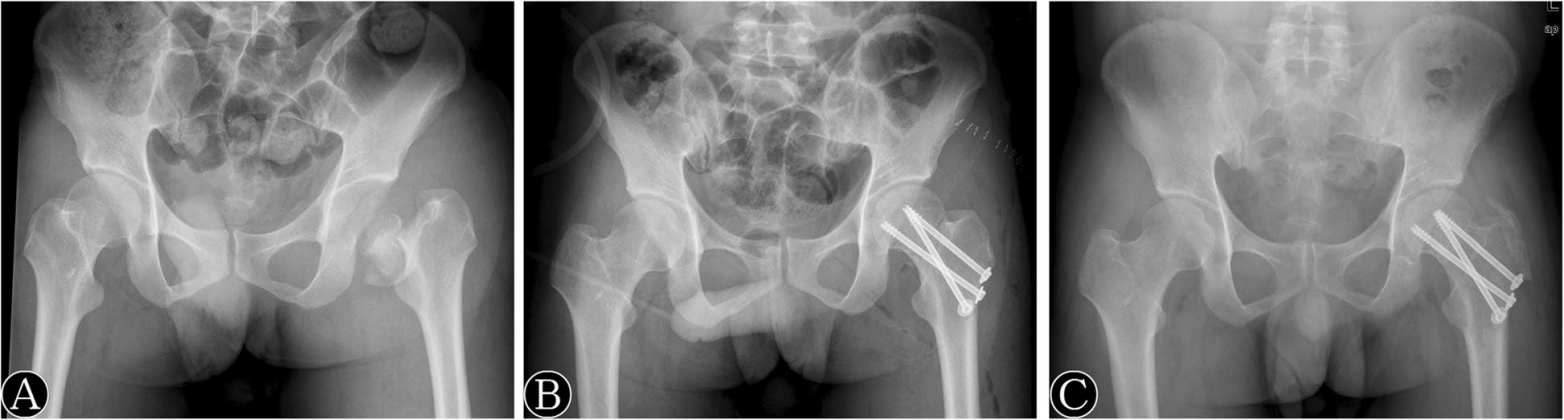
A-35-year-old man with left Pipkin type III fracture. **A**. Preoperative radiograph. **B**. Postoperative radiograph. **C**. Radiograph at 4 months demonstrating a bony union

At the final follow-up, the Thompson-Epstein criteria were excellent in 19 cases, good in 15 cases, fair in two cases, poor in three cases in early group and excellent in six cases, good in 15 cases, fair in six cases, poor in five cases in delayed group. The mean modified Merle D’Aubigné and Postel scores, VAS scores and PCS scores of patients in early group were higher than those in delayed group. There were statistically significant differences between the two groups in the Thompson-Epstein criteria, modified Merle D’Aubigné and Postel scores and PCS scores (*P* = 0.033, *P* = 0.010, *P* = 0.003, respectively) (Table 3). Series radiographs of typical cases are shown in Figs. 1, 2.

**Table 3:** Comparison of function evaluation between the two groups

	Early group (*n* = 39)	Delayed group (*n* = 32)	*p* value
Thompson-Epstein criteria			0.033
Excellent	19	6	
Good	15	15	
Fair	2	6	
Poor	3	5	
Modified Merle D’Aubigné and Postel scores	16.1 ± 2.7	14.2 ± 3.6	0.010
VAS	1.7 ± 2.0	2.6 ± 2.7	0.117
SF-12			
PCS	75.4 ± 24.1	58.8 ± 29.9	0.003
MCS	74.6 ± 10.4	69.1 ± 12.5	0.050

*VAS* visual analog scale, *SF-12* Medical Outcomes Short Form 12-item questionnaire score, *MCS* mental component scale, *PCS* physical component scale

## Discussion

The main injury mechanism of Pipkin fracture-dislocations is traumatic posterior hip dislocation, and the early and prompt hip reduction is particularly important [1–3]. Generally speaking, in the absence of contraindications, closed reduction under anesthesia or sedation in the operating room is the most commonly used method [1, 22]. In recent years, more and more evidences show that urgent closed reduction in the Emergency Department is also an effective and safe method [1, 23]. If the closed reduction fails or the concentric reduction cannot be achieved, the open reduction should be performed immediately.

While lots of evidences suggested that the reduction timing of hip for femoral fracture-dislocation is critical, there is still no consensus on the safe interval time between injury and hip reduction, and the reduction timing of posterior hip reduction is different in previous studies [22, 24]. Early research suggested that the posterior hip dislocation should be reduced within 24 h, otherwise the prognosis will be poor [16]. However, more and more studies reported that hip reduction within 6 h is helpful to minimize the incidence of complications and achieve a good result [17, 24]. There is also evidence that good results were achieved when hip reduction was performed within 12 h [15].

In our study, we found that the early reduction of posterior hip dislocation within 6 h is beneficial to fracture healing. Femoral head fractures are often accompanied by severe bone and soft tissue damage. Prolonged posterior dislocation of hip will cause vasospasm, which further damage the blood supply of the femoral head and affect the healing of fractures [1, 2, 11, 22, 24]. Early reduction of dislocation may restore the blood supply to the fractures site by relieving tension across the femoral and circumflex vessel, which is beneficial to fracture healing [24]. Besides, our research also revealed that the early hip reduction could shorten the mean length of hospital stay. There might be several reasons for this. On one hand, the severe damage of soft tissue and blood supply caused by prolonged hip dislocation may affect the healing of incision, thereby prolonging the length of hospital. On the other hand, the prolonged dislocation of hip will aggravate the swelling of soft tissue and affect the recovery of limb function.

The common complications of femoral head fractures include post-traumatic osteoarthritis, HO, ANFH and nonunion. The incidence of post-traumatic osteoarthritis is approximately 20%, which is related to the reduction timing of hip dislocation and the quality of fracture reduction [18]. Our data show a lower incidence of post-traumatic osteoarthritis when hip reduction is performed within 6 h. Similarly, prolonged dislocation of hip joint may be associated with a higher rate of ANFH [11, 13, 22]. Mehlman et al. [25] reported that patients whose hip reduction was delayed greater than 6 h had a 20 times higher risk of having avascular necrosis develop compared with patients whose hips were reduced in 6 h or less. A meta-analysis showed that early reduction of posterior hip dislocation within 6 h have a lower rate of osteonecrosis of the femoral head compare with delayed reduction (over 6 h from the time of injury) [24]. Our study also emphasizes this point once again. At the final follow up, the function evaluation showed that a better functional outcome can be obtained in the early group.

There are several limitations to our study. One of the limitations is that this was a retrospective study. Second, our data was based on the clinical records, there may be some margins of error in the exact time from injury to hip reduction. Furthermore, the length of follow up is relatively insufficient, the longer follow-up was needed to further assess the long-term effects of reduction timing of posterior hip dislocation in treating femoral head fractures combined with posterior hip dislocation. We also found that the main reason for delayed reduction was the untimely transportation of patients, therefore it is necessary to develop and improve the transport systems so as to transport patients to the nearest trauma center immediately. This study is the first report to compare the outcome of early and delayed hip reduction on the surgical treatment of femoral head fracture combined with posterior hip dislocation.

## Conclusion

In summary, in patients with femoral head fractures combined with posterior hip dislocation, the early and prompt hip reduction within 6 h can effectively facilitate the fracture healing and patient rehabilitation, and obtain a better functional outcome.

## Data Availability

The datasets used and/or analyzed during the current study are available from the corresponding author on reasonable request.
